# Could circulating biomarkers of nitrosative stress and protein glycoxidation be useful in patients with gastric cancer?

**DOI:** 10.3389/fonc.2023.1213802

**Published:** 2023-07-12

**Authors:** Justyna Dorf, Anna Pryczynicz, Joanna Matowicka-Karna, Konrad Zaręba, Piotr Żukowski, Anna Zalewska, Mateusz Maciejczyk

**Affiliations:** ^1^ Department of Clinical Laboratory Diagnostics, Medical University of Bialystok, Bialystok, Poland; ^2^ Department of General Pathomorphology, Medical University of Bialystok, Bialystok, Poland; ^3^ 2^nd^ Clinical Department of General and Gastroenterological Surgery, Medical University of Bialystok, Bialystok, Poland; ^4^ Department of Restorative Dentistry, Croydon University Hospital, Croydon, United Kingdom; ^5^ Independent Laboratory of Experimental Dentistry, Medical University of Bialystok, Bialystok, Poland; ^6^ Department of Hygiene, Epidemiology and Ergonomics, Medical University of Bialystok, Bialystok, Poland

**Keywords:** gastric cancer, reactive nitrogen species, nitric oxide, glycoxidation, nitrosative and oxidative stress

## Abstract

**Background:**

Nitrosative stress leads to protein glycoxidation, but both processes may be strongly related to the cancer development. Therefore, the aim of this study was to assess the nitrosative stress and protein glycoxidation products in patients with gastric cancer in comparison with healthy controls. We are also the first to evaluate the diagnostic utility of nitrosative stress and protein glycoxidation markers in gastric cancer patients in respect to histopathological classifications (TNM, Lauren’s and Goseki’s classification) and histopathological parameters such as histological type, histological differentiation grade, presence of vascular or neural invasion, desmoplasia and *Helicobacter pylori* infection.

**Methods:**

The study included 50 patients with gastric cancer and 50 healthy controls matched for sex and age. Nitrosative stress parameters and protein glycoxidation products were measured colorimetrically/fluorometrically in plasma or serum samples. Student’s t-test or Mann-Whitney U-test were used for statistical analysis.

**Results:**

NO, S-nitrosothiols, nitrotyrosine, kynurenine, N-formylkynurenine, dityrosine, AGE and Amadori products were significantly increased whereas tryptophan fluorescence was decreased in patients with gastric cancer compared to the healthy control. Nitrosative stress and glycoxidation products may be useful in diagnosis of gastric cancer because they differentiate patients with gastric cancer from healthy individuals with high sensitivity and specificity. Some of the determined parameters are characterised by high AUC value in differentiation of GC patients according to the histopathological parameters.

**Conclusions:**

Gastric cancer is associated with enhanced circulating nitrosative stress and protein glycation. Although further research on a tissue model is needed, plasma/serum biomarkers may be dependent on tumour size, histological type, tumour invasion depth, presence of lymph node and distant metastasis, vascular and neural invasion and Helicobacter pylori infection. Thus, circulating biomarkers of nitrosative stress/protein glycoxidation may have potential diagnostic significance in gastric cancer patients.

## Introduction

Gastrointestinal tumours still remain one of the most common and aggressive types of neoplasms. The incidence of GI malignancies in 2018 among all cancers was 26.3% whereas percentage of mortality was 35.4% (1). Researchers from the International Agency for Research on Cancer (IARC) predict that the annual burden of gastric cancer will increase by about 63% of new cases and 66% of deaths by 2040 compared with 2020 (2). This may be associated with an increase in the incidence of this cancer in young people under 40 (3). Gastric cancer in young people still is a challenge because it is characterised by a highly aggressive growth pattern and a more advanced stage at diagnosis. Hence, early detection and treatment of gastric cancer may not only prevent further progression of an invasive cancer but also significantly reduce the percentage of patients mortality. Therefore, it is important to understand the biology of gastric cancer and explore new diagnostic biomarkers which could enable its early detection.

Numerous studies have identified *Helicobacter pylori* as the major risk factor in gastric carcinogenesis. *Helicobacter pylori* may synthesise several virulence factors which enable immune escape, colonise and induce disease (lipopolysaccharides (LPS), flagellum, VacA, BabA, and DupA) (4). These virulence factors may influence the balance between cell proliferation and apoptosis, which is an important element for the occurrence and development of GC. It has been discovered that CagA is linked to cell adhesion and proliferation in gastric epithelial cells, as well as oxidative and nitrosative stress development (4). Patients with *Helicobacter pylori* infection are characterised by increased expression of NADPH oxidase NOX and inducible nitric oxide synthase (iNOS) which are enzymes responsible for reactive oxygen (ROS) and nitrogen (RNS) species production (5). Production of ROS and RNS in gastric cells may be stimulated not only by presence of *Helicobacter pylori* but also exposure of gastric mucosa to environmental factors such as ultraviolet (UV) radiation, ingestion of nonsteroidal anti-inflammatory drugs (NSAIDs), cigarette smoking, alcohol consumption and many other exogenous agents (6). These factors can trigger inflammation by activating the epithelial cells, polymorphonuclear neutrophils and macrophages to generate inflammatory cytokines and other mediators that further contribute to oxidative and nitrosative stress. The most reactive nitrogen species is nitric oxide (NO). The effects of NO in malignant transformation are broad and include cellular transformation, formation of neoplastic lesions or initiation and regulation of the metastatic cascade and angiogenesis (7, 8). NO reacts with its intracellular environment to form other reactive metabolites including peroxynitrite, nitrite, nitrate, S-nitrosothiols and nitrotyrosine that induce genotoxic effects leading to DNA damage (9). Uncontrolled production of ROS and RNS and an ineffective antioxidant barrier may lead to oxidative damages to cellular components (10). Proteins are very sensitive to oxidation and glycation processes, and their combinational effect is often called glycoxidation (11). Glycoxidation causes protein denaturation, fragmentation, aggregation, and alteration/loss of biological function which may result in activation of multiple signalling pathways e.g. Nf-κB, initiation of inflammatory processes or apoptosis. One of most common biomarkers of glycoxidation are Amadori products and AGE which act by binding to RAGE (AGE-specific receptor). Research performed on breast cancer cell lines and patients with breast and ovarian cancer demonstrated that AGE-RAGE complex promotes tumour development, migration and metastasis (12–14). Tryptophan and its metabolites such as N-formylkynurenine, kynurenine and dityrosine are also considered as oxidation products. Studies on cell lines demonstrated that imbalances in tryptophan metabolism can have an important role in cancer, promoting tumour progression by suppressing antitumour immune responses and increasing the malignant properties of cancer cells (15). Moreover, tryptophan metabolites can potently promote cancer cell motility and metastasis (15).

However, there are no studies focusing on the glycoxidation products and their role in gastric carcinogenesis. A thorough understanding of the pathogenesis of gastric cancer may help to develop new therapeutic strategies or diagnostic methods, in order to detect the disease early on. In the available literature, there are only very few studies on the nitrosative stress and protein glycoxidation in patients with gastric cancer. Therefore, this case-control study aims to evaluate serum and plasma levels of nitrosative stress biomarkers (nitric oxide, nitrosothiols, nitrotyrosine) and protein glycoxidation products (tryptophan, kynurenine, N-formylkynurenine, dityrosine, Amadori products and advanced glycation end products (AGEs)) in 50 patients with gastric cancer in comparison with healthy controls. We also assess the diagnostic utility of these parameters using receiver operating characteristic (ROC) in respect to histopathological parameters such as TNM, Lauren’s and Goseki’s classifications, histological type and histological differentiation grade of the tumour, presence of *Helicobacter pylori* infection as well as presence of vascular or neural invasion and desmoplasia. We also evaluated a correlation between nitrosative stress parameters and proteins glycoxidation products and some chosen laboratory test results.

## Materials and methods

### Ethical statement

The study was approved by the Bioethics Committee of the Medical University of Bialystok, Poland (permission number APK-002/238/2022). The study was conducted in accordance with the World Medical Association Declaration of Helsinki for ethical principles for medical research involving human subjects. All patients gave their informed consent to participate in the study.

### Study group

The study group consisted of 50 patients who were treated surgically due to gastric cancer in the 2nd Clinical Department of General and Gastroenterological Surgery at the Medical University of Bialystok Clinical Hospital in 2017–2020. The patients qualified for the research were patients diagnosed with cancer at any stage who had not received radio- or chemotherapy prior to surgery; patients without squamous cell carcinoma and other non-epithelial neoplasms, metastases of other neoplasms to the stomach, acute inflammatory diseases, autoimmune and infectious diseases (HIV/AIDS, hepatitis, Crohn’s and Hashimoto’s disease, ulcerative colitis, rheumatoid arthritis and psoriasis), cardiovascular and metabolic diseases, such as osteoporosis, type 1 diabetes, mucopolysaccharidosis and gout, digestive, respiratory or genitourinary systems diseases. Additionally, smokers and patients taking drugs (glucocorticosteroids, non-steroidal anti-inflammatory drugs, antibiotics, vitamins, and dietary supplements) for the three months before the surgery were excluded from the study. Lack of complete medical documentation was also an exclusion criterion.

The time from cancer diagnosis to the surgical resection of the tumour ranged from two days to four weeks. The study material was taken from all patients before surgery.

### Control group

The control group included 50 healthy patients (selected by sex and age to match the study group) attending follow-up visits at the Department of Restorative Dentistry at the Medical University of Bialystok from January 2018 to January 2020. To the control group were qualified patients with normal results of complete blood count (CBC) and biochemical blood tests (AST, ALT, Na+, K+, creatinine).

### Histopathological analysis

In a routine histopathological examination we evaluated the histological type of tumour, histopathological grade (G) according to the World Health Organization (WHO) guidelines (16), depth of tumour invasion (pT), presence of lymph node metastasis (pN) and distant metastasis (pM) according to the TNM classification standard of the Union for International Cancer Control (17), type of cancer according to Lauren (18) and Goseki (19) classification, vascular and neural invasion and degree of desmoplasia. Moreover, *Helicobacter pylori* infection was assessed in Giemsa-stained preparations.

Tissue sections taken during the surgery were fixed in 10% buffered formalin solution and embedded in paraffin at a temperature of 56°C. The paraffin blocks were then sliced with a microtome (Microm H340) into approximately 4 μm-thick slides and stained with hematoxylin and eosin. The obtained sections were reviewed by two independent pathologists on a microscope Olympus CX22 under 200× and 400× magnification.

### Blood collection

All samples were collected from patients in a fasting state both with gastric cancer and healthy individuals who did not perform intense physical exercise twenty-four hours prior to blood sampling. Blood was collected into K3-EDTA and clotting activator tube (S-Monovette SARSTEDT) and centrifuged at 4000 rpm for 10 minutes at 4°C (MPW 351, MPW Med. Instruments, Warsaw, Poland) to separate plasma or serum from erythrocytes. The top layer (plasma or serum) was then taken and was protected against oxidation (10 μl of 0.5M BHT/1 ml of serum/plasma) and stored at -80°C until the final analysis. Until redox determinations, all samples were stored at -80°C for no longer than six months.

### Redox assays

All reagents (unless otherwise stated) were analytical grade and purchased from Sigma-Aldrich Nümbrecht (Germany) or Sigma-Aldrich Saint Louis (MO, USA). The 96-well microplate reader BioTek Synergy H1 (Winooski, VT, USA) was used to measure absorbance/fluorescence. All assays were performed in duplicate samples and standardised to 1 mg of the total protein. The total protein content was determined spectrophotometrically (Thermo Scientific PIERCE BCA Protein Assay; Rockford, IL, USA).

### Nitrosative stress

#### Nitric oxide (NO)

Nitric oxide (NO) concentration was evaluated indirectly through determination of its stable decomposition products NO_2_^-^ and NO_3_^-^ using the Griess reaction. Briefly, 100 μL of samples was incubated at 37°C for 15 min (500 rpm) with 100 μL of freshly prepared Griess reagent (1% sulfanilamide and 0.1% NEDA · 2 HCl(N-(1-naphthyl)-ethylenediamine dihydrochloride in 2.5% metaphocphoric acid). The absorbance of 96-well microplates was analysed at 490 nm. The NO level was calculated from the calibration curve for NaNO_2_ (20, 21).

#### S-nitrosothiols

The concentration of S-nitrosothiols was analysed spectrophotometrically using the Griess’s reaction with Cu^2+^ ions (22). Briefly, 10 μL of samples was incubated at 37°C for 20 min (500 rpm) with 190 μL of freshly prepared modified Griess reagent (1% sulfanilamide, 0.1% mM NEDA · 2 HCl(N-(1-naphthyl)-ethylenediamine dihydrochloride and 5% CuCl_2_ in phosphate buffered saline, pH 7.4). The absorbance was assessed at 490 nm with the use of extinction coefficient ε=11 500 M^- 1^cm^-1^.

#### Nitrotyrosine

The concentration of nitrotyrosine was determined spectrophotometrically by ELISA method. Commercial diagnostic kit (Immundiagnostik AG; Bensheim, Germany) was used according to the manufacturer’s instructions.

### Protein glycoxidation products

#### Tryptophan, kynurenine, N-formylkynurenine, dityrosine

To evaluate the protein glycoxidation rate, the characteristic fluorescence emission and excitation at 295/340 nm (tryptophan), 365/480 nm (kynurenine), 325/434 nm (N-formylkynurenine), and 330/415 nm (dityrosine) was measured. Prior to the determination, samples were diluted in 0.1 M sulphuric acid (1:5, v/v) (23–25). Next, the characteristic fluorescence was measured in 200 μL of diluted samples applied on 96-well black-bottom microplates. The results were expressed in arbitrary fluorescence units (AFU)/mg protein.

#### Amadori products

The content of the Amadori product was determined colorimetrically using nitro blue tetrazolium (NBT) assay (25). Briefly, 100 μL of samples was incubated at 37°C for 2 h (500 rpm) with 100 μL of NBT reagent (250 μM NBT in 0.1 M carbonate buffer, pH 10.35). The absorbance of 96-well microplates was measured at 525 nm with the use of monoformazan extinction coefficient (12,640 M^-1^cm^-1^).

#### Advanced glycation end products

The content of plasma advanced glycation end products (AGE) was measured spectrofluorimetrically. The characteristic fluorescence of pyraline, pentosidine, furyl-furanyl-imidazole (FFI), and carboxymethyl lysine (CML) was assessed at 350/440 nm by measuring AGE specific fluorescence (26). Prior to the determination, samples were diluted in 0.1 M sulphuric acid (1:5, v/v) (20, 21). Next, characteristic fluorescence was measured in 200 μL of diluted samples applied on 96-well black-bottom microplates.

### Statistical analysis

The statistical significance level was established at p < 0.05. The normality of the distribution was evaluated using the Shapiro–Wilk test, while homogeneity of variance used the Levene test. In order to compare the differences between two-group with large number of variables multivariate permutational test has been performed. Then, to compare differences between two independent groups with the lack of normal distribution, the Mann-Whitney U test was used. The results were presented as median (minimum-maximum). The relationship between the assessed redox biomarkers and clinicopathological parameters was assessed using the Spearman rank correlation. In order to identify factors that determine the levels of redox biomarkers, we performed multiple regression analyses. Histological differentiation grade, pT, pN, and pM were included as independent variables; 95% confidence intervals (CI) were noted along with regression parameters. Receiver Operating Characteristic (ROC) analysis was used to assess the diagnostic utility of the redox biomarkers. AUC (area under the curve) and optimal cut-off values were determined for each parameter, ensuring high sensitivity and specificity.

The number of subjects was based on our previous experiment involving 15 patients (online ClinCalc software). The variables used for the sample size calculations were concentrations of NO and AGE. The level of significance was set at 0.05 and power of study was 0.9. The ClinCalc sample size calculator provided the sample size for one group. The minimum number of patients was 38.

Statistical analysis was performed using GraphPad Prism 9.0 (GraphPad Software, La Jolla, USA) and Past 4.13 (Øyvind Hammer).

## Results

### Characteristics of the study group

The study included 50 patients (33 male, 17 female) with gastric cancer and 50 healthy people (28 male, 22 female) at age 40-85. 28% of patients with gastric cancer had a tumour with a diameter ≤5 cm, whereas 72% had a tumour of a diameter greater than 5 cm. 68% of patients had adenocarcinoma whereas 32% had adenocarcinoma mucinosum. G2 grade of the tumour was observed in 40% of patients whereas G3 was present in 60%. *Helicobacter pylori* infection was confirmed in 36% of patients. 40% of patients had a pT1 or pT2 stage of tumour and 60% had pT3 and pT4 stage. 76% of patients had lymph node (N1+N2) and 34% had distant metastasis (M1+M2). 76% of patients had a vascular invasion and 72% of patients had neural invasion. Small desmoplasia was present in 70% of patients and diffuse in 30%. Diffuse type of gastric cancer was observed in 58% of patients whereas intestinal type in 42%. I and II type of GC according to Goseki classification was present in 50% of patients and III and IV in 50% of patients. 80% of patients had a normal level of CEA (<5.0 ng/ml) and CA72-4 (<6.9 U/ml) and 75.5% had a normal level of CA19-9 (<35 U/ml). Detailed patient characteristics are summarised in **Table 1**.

**Table 1:** Characteristics of study group.

Parameter	n (%)
Age	
<60	10 (20.0%)
>60	40 (80.0%)
Sex	
male	33 (66.0%)
female	17 (34.0%)
Tumour’s diameter	
≤5cm	14 (28.0%)
>5cm	36 (72.0%)
Tumor location	
Upper 1/3	17 (34.0%)
Middle 1/3	15 (30.0%)
Lower 1/3	10 (20.0%)
Whole stomach	8 (16.0%)
Histological type	
adenocarcinoma	34 (68.0%)
adenocarcinoma mucinosum	16 (32.0%)
Histological differentiation grade	
moderately differentiated	20 (40.0%)
poorly differentiated	30 (60.0%)
Helicobacter pylori	
absent	32 (64.0%)
present	18 (36.0%)
Depth of invasion	
T1+T2	20 (40.0%)
T3+T4	30 (60.0%)
Lymph node metastasis	
N0	12 (24.0%)
N1+N2	38 (76.0%)
Distant metastasis	
M0	33 (66.0%)
M1+M2	17 (34.0%)
Vascular invasion	
absent	12 (24.0%)
present	38 (76.0%)
Neural invasion	
absent	14 (28.0%)
present	36 (72.0%)
Desmoplasia	
small	35 (70.0%)
diffuse	15 (30.0%)
Lauren’s classification	
intestinal	21 (42.0%)
diffuse	29 (58.0%)
Goseki classification	
I+II	25 (50.0%)
III+IV	25 (50.0%)
CEA level (ng/ml)	
0-5.0	40 (80.0%)
>5.0	10 (20.0%)
CA 19-9 (U/ml)	
0-35	39 (75.5%)
>35	11 (24.5%)
CA 72-4 (U/ml)	
0 – 6.9	40 (80.0%)
>6.9	10 (20.0%)
IL-6 (pg/ml)	
0-7.0	7 (14.0%)
>7.0	43 (86.0%)

CEA, carcinoembryonic antigen, CA19-9, carbohydrate antigen 19-9; CA72-4, carbohydrate antigen 72-4; IL-6, interleukin 6.

### Comparison of nitrosative stress and glycoxidation products between patients with gastric cancer and healthy controls

**Figure 1:**
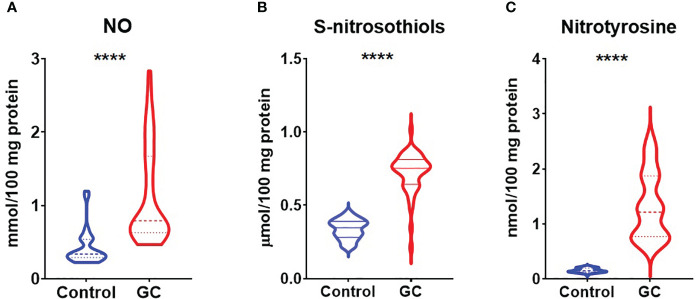
Comparison of nitrosative stress parameters – NO **(A)**, S-nitrosothiols **(B)** and nitrotyrosine **(C)** between patients with gastric cancer and the control group. The data are presented as median (minimum - maximum). ****p<0.0001. GC, gastric cancer; NO, nitric oxide.

**Figure 2:**
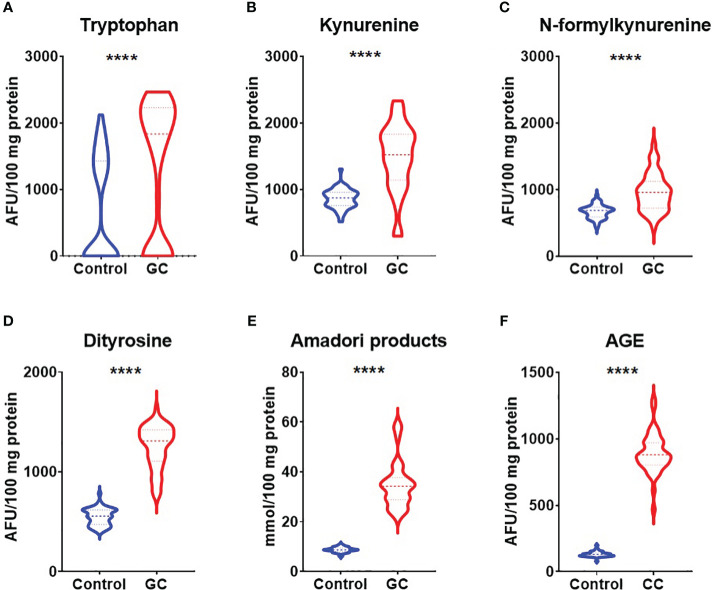
Comparison of glycoxidation products – tryptophan **(A)**, kynurenine **(B)**, N-formylkynurenine **(C)**, dityrosine **(D)**, Amadori products **(E)** and AGE **(F)** between patients with gastric cancer and the control group. The data are presented as median (minimum - maximum). ****p<0.0001. AGE, advanced glycation end products; GC, gastric cancer.

In order to evaluate nitrosative stress we analysed concentrations of NO, S-nitrosothiols and nitrotyrosine. Concentrations of NO, S-nitrosothiols and nitrotyrosine were considerably higher in patients with gastric cancer than in healthy controls (p<0.0001, p<0.0001, p<0.0001) (**Figures 1A-C**). We also assessed concentrations of tryptophan, kynurenine, N-formylkynurenine, dityrosine, Amadori products and AGE which are protein glycoxidation products. Generally, the fluorescence of kynurenine, N-formylkynurenine, dityrosine, Amadori products, AGE were significantly higher (p<0.0001, p<0.0001, p<0.0001, p<0.0001, p<0.0001) whereas tryptophan level was significantly lower (p<0.0001) in patients with gastric cancer in comparison to the healthy control (**Figures 2A-F**).

### Comparison of nitrosative stress and glycoxidation products between women and men with gastric cancer

We also compared concentrations of NO, S-nitrosothiols and nitrotyrosine as well as concentrations of tryptophan, kynurenine, N-formylkynurenine, dityrosine, Amadori products and AGE between groups of women and men with gastric cancer but we didn’t observe statistically significant differences (**Supplementary File 1**).

### Comparison of nitrosative stress in groups of patients with gastric cancer according the chosen histopathological parameters

**Figure 3:**
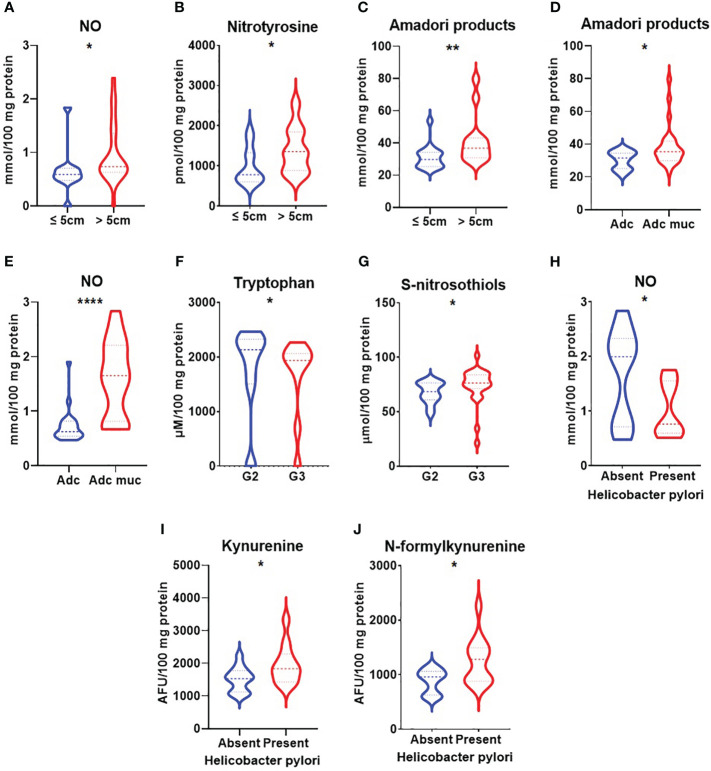
Comparison of NO **(A)**, nitrotyrosine **(B)**, Amadori products **(C)** concentration between patients with tumours diameter ≤5cm and >5cm; Amadori products **(D)** and NO **(E)** level between patients with adenocarcinoma and adenocarcinoma mucinosum; tryptophan **(F)**, and S-nitrosothiols **(G)** between patients with tumours in G2 and G3 stage; NO **(H)**, kynurenine **(I)** and N-formylkynurenine **(J)** between patients with absent and present *Helicobacter pylori* infection. Adc, adenocarcinoma; Adc muc, adenocarcinoma mucinosum; NO, nitric oxide. *p < 0.05, **p < 0.01, ****p<0.0001.

**Figure 4:**
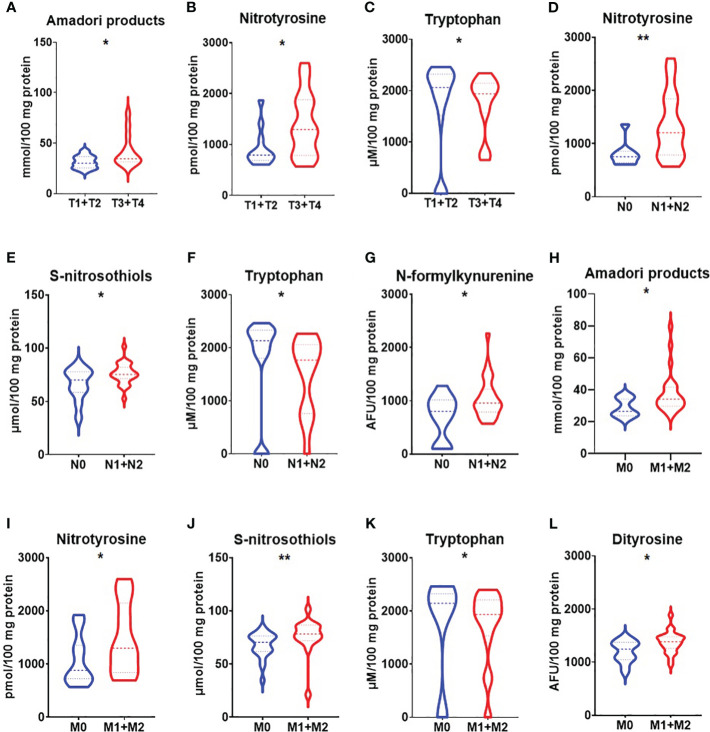
Comparison of Amadori products **(A)**, nitrotyrosine **(B)** and tryptophan **(C)** between patients with tumours in T1 and T2 stage vs T3 and T4 stage; nitrotyrosine **(D)**, S-nitrosothiols **(E)**, tryptophan **(F)** and N-formylkynurenine **(G)** between groups of patients without and with lymph node metastasis; Amadori products **(H)**, nitrotyrosine **(I)**, S-nitrosothiols **(J)**, tryptophan **(K)** and dityrosine **(L)** between groups of patients without and with distant metastasis. The data are presented as median (minimum - maximum). *p < 0.05, **p < 0.01.

**Figure 5:**
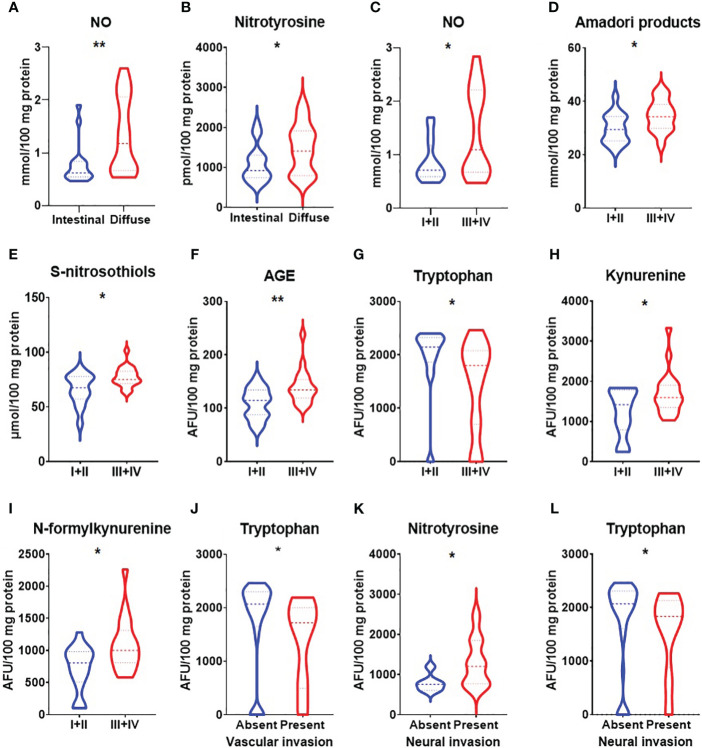
Comparison of NO **(A)** and nitrotyrosine **(B)** concentration between groups of patients with intestinal and diffuse types of gastric cancer according to Lauren classification; NO **(C)**, Amadori products **(D)**, S-nitrosothiols **(E)**, AGE **(F)**, tryptophan **(G)**, kynurenine **(H)** and N-formylkynurenine **(I)** between I+II and III+IV types of gastric cancer according to Goseki classification; tryptophan **(J)** between groups of patients with absent and present vascular invasion, nitrotyrosine **(K)** and tryptophan **(L)** between groups of patients with absent and present neural invasion. NO, nitric oxide; AGE, advanced glycation end products. The data are presented as median (minimum - maximum). *p < 0.05, **p < 0.01.

We demonstrated increased NO and nitrotyrosine concentrations in a group of patients with gastric cancer with a tumour diameter >5 cm (p<0.05, p<0.05) (**Figures 3A, B**). NO level was also significantly higher in patients with adenocarcinoma mucinosum and lower in patients with Helicobacter pylori infection (p<0.0001, p<0.05) (**Figures 3E, H**). S-nitrosothiols were increased in patients with poorly differentiated tumours in comparison with moderately differentiated tumours (p<0.05) (**Figure 3G**). There was a statistically significant increase in nitrotyrosine concentration in patients with T3+T4 stages (p<0.05), in patients with lymph node (p<0.01) and distant metastasis (p<0.05) (**Figures 4B, D, I**). S-nitrosothiols were considerably higher in patients with lymph node and distant metastasis than in patients without metastasis (p<0.05, p<0.01) (**Figures 4E, J**). Nitrotyrosine level was significantly higher in patients with diffuse type of GC and in patients with vascular infiltration (p<0.05, p<0.05) (**Figures 5B, K**). We also observed an increase of NO level in patients with diffuse type of GC compared to intestinal type of GC (p<0.01) as well as in patients in III and IV stages according to Goseki classification compared to I and II stages (p<0.05) (**Figures 5A, C, E**).

### Comparison of glycoxidation products in groups of patients with gastric cancer according the chosen histopathological parameters

Amadori products were considerably higher in patients with a tumour diameter >5cm (p<0.01) as well as in patients with adenocarcinoma mucinosum (p<0.05) (**Figures 3C, D**). We observed statistically significant higher tryptophan level in patients in G2 stage compared to G3 (<0.05) (**Figure 3F**). Kynurenine and N-formylkynurenine were significantly increased in patients with *Helicobacter pylori* infection in comparison to patients without infection (p<0.05, p<0.05) (**Figures 3I, J**). In patients with tumours in T3+T4 stages, Amadori products were statistically significant increased (p<0.05) whereas tryptophan level was decreased (p<0.05) compared to patients with tumours in T1+T2 stages (**Figures 4A, C**). We also observed statistically significant differences in tryptophan and N-formylkynurenine concentration between patients with and without lymph node metastasis (p<0.01, p<0.05) (**Figures 4F, G**). The fluorescence of Amadori products and dityrosine were considerably higher (p<0.05, p<0.05) whereas tryptophan was considerably lower (p<0.01) in patients with distant metastasis compared to those without metastasis (**Figures 4H, K, L**). We observed statistically significant higher levels of Amadori products, kynurenine and N-formylkynurenine (p<0.05, p<0.05, p<0.05) whereas tryptophan was lower (p<0.05) in patients in III and IV stages according to Goseki classification compared to I and II stages (**Figures 5D, F–I**). Interestingly, tryptophan level was also considerably higher in patients without vascular and neural infiltration compared to those without infiltration (p<0.05, 0<0.05) (**Figures 5J, L**).

### ROC analysis

In order to evaluate the diagnostic value of nitrosative stress and glycoxidation biomarkers in the diagnostics of gastric cancer we performed a ROC analysis (**Tables 2**–**5**). We showed high diagnostic values of all determined redox parameters in identification of patients with gastric cancer from healthy controls (**Table 2**). NO (AUC=0.75), nitrotyrosine (AUC=0.75) and Amadori products (AUC=0.78) may be useful in differentiation of patients according to the tumour size (**Table 3**). Amadori products (AUC=0.72) and NO (AUC=0.87) proved to be helpful in differentiation of patients with adenocarcinoma from adenocarcinoma mucinosum (**Table 3**). We showed high diagnostic values of S-nitrosothiols (AUC=0.72) and tryptophan (AUC=0.71) in recognition of patients with moderately differentiated (G2) from poorly differentiated tumours (G3) (**Table 3**). We also demonstrated very high diagnostic values of NO (AUC=0.76), kynurenine (AUC=0.74) and N-formylkynurenine (AUC=0.75) in differentiation of groups of patients with gastric cancer with present and absent *Helicobacter pylori* infection (**Table 3**). Amadori products (AUC=0.73), nitrotyrosine (AUC=0.73) and tryptophan (AUC=0.72) proved to be helpful in differentiation of patients with cancer at stages pT1+pT2 from patients with tumours at stages pT3+pT4 (**Table 4**). Parameters like nitrotyrosine (AUC=0.79), S-nitrosothiols (AUC=0.69), tryptophan (AUC=0.83) and N-formylkynurenine (AUC=0.68) may be valuable to help to diagnose patients with lymph node metastasis as oppose to those without lymph node metastasis (**Table 4**). Amadori products (AUC=0.76), nitrotyrosine (AUC=0.70), S-nitrosothiols (AUC=0.76), tryptophan (AUC=0.76) and dityrosine (AUC=0.73) showed a high diagnostic value in differentiation of patients with present and absent distant metastasis (**Table 4**). We also demonstrated a high diagnostic value of nitrotyrosine (AUC=0.68) and NO (AUC=0.76) in differentiation of diffuse type from intestinal type of gastric cancer according to Lauren classification and Amadori products (AUC=0.72), NO (AUC=0.71), S-nitrosothiols (AUC=0.73), AGE (AUC=0.76), tryptophan (AUC=0.73), kynurenine (AUC=0.69) and N-formylkynurenine (AUC=0.75) in differentiation of I+II types from III+IV types of gastric cancer according to Goseki classification (**Table 5**). Particular attention should be paid to tryptophan for which AUC in the presence of vascular invasion was 0.80 and nitrotyrosine and tryptophan with AUC=0.78, AUC=0.73 in the presence of neural invasion (**Table 5**).

**Table 2:** ROC analysis of nitrosative stress parameters and protein glycooxidation products between patients with gastric cancer and the healthy controls.

Parameter	AUC	p-value	Cut-off	Sensitivity (%)	Specificity (%)	95% confidence interval
Nitrosative stress						
NO	0.9160	<0.0001	> 0.5417	88.89	81.48	0.8433 to 0.9888
S-nitrosothiols	0.9659	<0.0001	> 0.4381	95.45	95.83	0.9167 to 1.000
Nitrotyrosine	1.000	<0.0001	> 401.3	100.0	100.0	1.000 to 1.000
Protein glycooxidation products						
Tryptophan	0.8651	<0.0001	> 17.64	80.95	80.00	0.7794 to 0.9507
Kynurenine	0.8833	<0.0001	> 982.9	86.05	84.00	0.7970 to 0.9695
N-formylkynurenine	0.8300	<0.0001	> 727.5	73.81	74.00	0.7400 to 0.9200
Dityrosine	0.9995	<0.0001	> 735.8	100.0	98.00	0.9980 to 1.000
Amadori products	1.000	<0.0001	> 16.19	100.0	100.0	1.000 to 1.000
AGE	1.000	<0.0001	> 333.9	100.0	100.0	1.000 to 1.000

AUC, area under curve; AGE, advanced glycation end products; NO, nitric oxide.

**Table 3:** Receiver operating characteristic (ROC) analysis in gastric cancer patients for different tumour size, histological type, histological differentiation grade and Helicobacter pylori infection.

Parameter	AUC	p-value	Cut-off	Sensitivity (%)	Specificity (%)	95% confidence interval
Tumour size						
NO	0.7510	0.0194	> 0.6859	65.22	72.73	0.5733 to 0.9287
Nitrotyrosine	0.7489	0.0225	> 1032	66.67	63.64	0.5633 to 0.9346
Amadori products	0.7861	0.0091	> 34.14	69.23	75.00	0.6192 to 0.9529
Histological type						
Amadori products	0.7164	0.0410	> 34.03	64.00	63.64	0.5414 to 0.8914
NO	0.8737	<0.0001	> 0.7832	78.95	75.00	0.7657 to 0.9816
Histological differentiation grade						
S-nitrosothiols	0.7229	0.0224	> 74.39	64.00	64.29	0.5645 to 0.8812
Tryptophan	0.7143	0.0273	> 20.66	66.67	67.85	0.5455 to 0.8831
Helicobacter pylori infection						
NO	0.7596	0.0276	> 1.400	61.54	66.67	0.5610 to 0.9582
Kynurenine	0.7403	0.0428	> 1674	72.73	71.43	0.5417 to 0.9388
N-formylkynurenine	0.7500	0.0339	> 1001	61.54	66.67	0.5550 to 0.9450

AUC, area under curve; NO, nitric oxide.

**Table 4:** Receiver operating characteristic (ROC) analysis in gastric cancer patients for different depth of tumour invasion, lymph node and distant metastasis.

Parameter	AUC	p-value	Cut-off	Sensitivity (%)	Specificity (%)	95% confidence interval
Depth of tumour invasion						
Amadori products	0.7308	0.0256	> 34.14	69.23	66.67	0.5604 to 0.9012
Nitrotyrosine	0.7346	0.0207	> 885.7	74.07	75.00	0.5701 to 0.8990
Tryptophan	0.7246	0.0206	> 20.81	60.87	66.67	0.5638 to 0.8855
Presence of lymph node metastasis						
Nitrotyrosine	0.7937	0.0088	> 857.1	71.43	77.78	0.6405 to 0.9468
S-nitrosothiols	0.6909	0.0414	> 74.39	60.00	54.55	0.4920 to 0.8898
Tryptophan	0.8255	0.0021	> 20.51	72.00	72.73	0.6831 to 0.9678
N-formylkynurenine	0.6853	0.082	> 863.8	65.38	63.64	0.4927 to 0.8780
Presence of distant metastasis						
Amadori products	0.7566	0.0157	> 30.78	66.67	63.64	0.5783 to 0.9369
Nitrotyrosine	0.7038	0.0322	> 1177	62.50	60.87	0.5397 to 0.8679
S-nitrosothiols	0.7571	0.0075	> 75.26	68.75	72.73	0.5950 to 0.9192
Tryptophan	0.7628	0.0091	> 21.39	69.23	66.67	0.6099 to 0.9157
Dityrosine	0.7267	0.0222	> 1313	65.22	64.29	0.5629 to 0.8905

AUC, area under curve.

**Table 5:** Receiver operating characteristic (ROC) analysis in gastric cancer patients for type of GC according to Lauren and Goseki classifications and vascular and neural invasion.

Parameter	AUC	p-value	Cut-off	Sensitivity (%)	Specificity (%)	95% confidence interval
Lauren classification						
Nitrotyrosine	0.6863	0.0499	> 1195	64.71	66.67	0.5056 to 0.8670
NO	0.7647	0.0067	> 0.7183	63.16	58.82	0.6092 to 0.9202
Goseki classification						
Amadori products	0.7235	0.0314	> 32.08	66.67	64.71	0.5462 to 0.9008
NO	0.7070	0.0407	> 0.7957	57.89	60.00	0.5316 to 0.8824
S-nitrosothiols	0.7330	0.0211	> 74.39	61.90	57.14	0.5560 to 0.9100
AGE	0.7582	0.0091	> 123.1	66.67	64.71	0.5985 to 0.9179
Tryptophan	0.7276	0.0199	> 20.68	63.16	64.71	0.5581 to 0.8970
Kynurenine	0.6974	0.0469	> 1476	68.42	62.50	0.5192 to 0.8755
N-formylkynurenine	0.7461	0.0118	> 928.5	63.16	64.71	0.5873 to 0.9049
Vascular invasion						
Tryptophan	0.8000	0.0334	> 19.86	70.97	80.00	0.6102 to 0.9898
Neural invasion						
Nitrotyrosine	0.7865	0.0277	> 817.0	68.75	66.67	0.6195 to 0.9534
Tryptophan	0.7333	0.0474	> 19.86	70.00	71.43	0.5401 to 0.9266

AUC, area under curve; AGE, advanced glycation end products; NO, nitric oxide.

## Discussion

Oxidative/nitrosative stress is associated with the development of gastrointestinal cancers including colorectal cancer (27–30), liver (31), oesophagus (32), and pancreatic cancers (33). However, the exact role of ROS/RNS in gastric carcinogenesis is still unclear and unexplained. In our previous published paper, we demonstrated that colorectal cancer is strongly associated with enhanced oxidative stress and increased nitrosative damages to proteins and DNA. These parameters differ between cancerous and non-cancerous tissue as well as tumours located in the right- and left side of the colon. We showed that assessment of nitrosative stress parameters could be helpful for evaluating the progression and differentiation of the tumour location. We also showed that redox parameters may be associated with histological type of the tumour and may influence tumour invasion depth, presence of lymph node and distant metastasis, vascular and neural invasion, inflammatory invasion, and tumour budding, which are part of the tumour microenvironment. These parameters are considered as independent adverse prognostic factors in patients with primary operable colorectal cancer (29). Therefore, we decided to assess the nitrosative stress and glycoxidation products in patients with gastric cancer. We observed increased levels of NO, nitrotyrosine, S-nitrosothiols, kynurenine, N-formylkynurenine, dityrosine, AGE and Amadori products and decreased concentration of tryptophan in GC patients in comparison with the control group. We observed significantly differences in nitrosative stress and glycoxidation products according to tumour size, histological type and histological differentiation grade of the tumour, depth of tumour invasion, presence of lymph node and distant metastasis, presence of *Helicobacter pylori* infection, Lauren and Goseki classification, as well as presence of vascular or neural invasion and desmoplasia. Some of the determined parameters showed high diagnostic value in diagnosis of gastric cancer as well as differentiation of patients according to histopathological parameters.

### Nitrosative stress in patients with gastric cancer

The gastric mucosa is a barrier protecting deeper tissues from the gastric juice and external oxidants such as ethanol, cigarette smoking or nonsteroidal anti-inflammatory drugs. Exposure of gastric mucosa to environmental factors may result in enhanced production of ROS/RNS, which consequently induces oxidative and nitrosative stress. Redox imbalance in the stomach may be caused by decreased activity of antioxidant enzymes – catalase, glutathione peroxidase, glutathione reductase and reduced glutathione, which we demonstrated in our previous papers (27, 28). Among the RNS, the major player is nitric oxide which is produced by nitric oxide synthases (NOS) from L-arginine and oxygen (34). Studies performed on cell lines demonstrated that NO may have dual effects in cancer – may promote tumour growth and proliferation. On the other hand NO presents with tumouricidal effects however there is lack of data of its activity in cancer patients (7). NO action depends on its location and concentration. At lower concentrations, NO supports cancer development, while at high concentrations are cytotoxic to cancerous cells and induces apoptosis by forming peroxynitrite (35). Therefore it is not surprising that we observed significantly higher NO concentration in patients with gastric cancer. We also observed a significantly lower NO level in GC patients with *Helicobacter pylori* infection compared to those without infection. *Helicobacter pylori* may produce a large amount of superoxide anion to inhibit the bactericidal effects of nitric oxide generated by inflammatory cells in the stomach (36). The decrease in NO concentration in GC patients with *Helicobacter pylori* infection may be associated with effective scavenging of NO (37). In these patients, NO production may be suppressed by H. pylori which induces apoptosis of macrophages (36). NO levels were increased in patients with diffuse type of GC according to Lauren classification and in III+IV type of GC according to Goseki classification. These patients present with a worse overall survival rate than intestinal and I and II types of gastric cancer (38, 39).

Enhanced production of NO and other RNS may result in posttranslational modifications of proteins such as S-nitrosylation, glutathionylation and tyrosine nitration. S-nitrosylation is defined as a selective covalent post-translational modification that adds a nitrosyl group to the crucial thiol/sulfhydryl of cysteine to form an S-nitrosothiol (RSNO) derivative. The S-nitrosylation mechanism shows a protective action in different systems, preventing some critical protein thiols from further irreversible modifications by reactive oxygen species. However, the S-nitrosothiols produced in this reaction can alter the cellular function of several proteins (34). Dysregulated S-nitrosylation may take part in malignant transformation through genomic instability, cell proliferation, apoptosis inhibition, angiogenesis, and metabolic reprogramming (40). In our study we observed significantly increased levels of S-nitrosothiols in gastric cancer patients. S-nitrosothiols were also increased in patients in G3 stage as well as in patients with lymph node and distant metastasis. Similar observations were made by Ehrenfald et al. in breast cancer (41). They concluded that S-nitrosylation promotes tumour cell epithelial-to-mesenchymal transition (EMT) and facilitates migration and invasion by promoting adhesion to the endothelium and intravasation and extravasation (41). Increased values of S-nitrosothiols were observed in III+IV types of gastric cancer according to Goseki classification in comparison with I+II. It may be suggested that S-nitrosylation may be associated with development of a more aggressive type of cancer. This was confirmed in studies conducted in patients with breast cancer, which showed that increased S-nitrosylation leads to the activation of Ets-1 caused by MAPK-dependent phosphorylation and results in development of more aggressive breast cancer phenotype [41]. Moreover, the latest reports indicate that anti-cancer strategies should reduce S-nitrosylation. This can be achieved by using NOS inhibitors, NO scavengers, or denitrosylase mimetics (40).

As mentioned above, RNS may also induce a two-step reaction of tyrosine nitration (42). The presence of nitrotyrosine in proteins indicates a high intensity of nitrosative modifications of proteins that favour pro-oxidant processes (43). In addition, nitration of tyrosine residues is considered as a biomarker for ONOO^-^, which is generated by the reaction of NO with O2^-^. This is due to the fact that ONOO^-^ is generally more reactive and toxic than NO. Moreover, ONOO- is a powerful biooxidant and reacts directly with sulfhydryls, iron–sulphur, and zinc–thiolate groups and participates in oxidation and hydroxylation reactions (44). Protein nitration occurs under physiological conditions and is responsible for regulation of multiple biological processes such as energy metabolism, signal transduction, enzyme inactivation, protein degradation, mitochondrial dysfunction, immunogenic response, apoptosis, and cell death (45). In our work we observed higher levels of nitrotyrosine in gastric cancer patients compared to the healthy control. It indicates that excessive or incorrect nitration and accumulation of nitro‐modified proteins like nitrotyrosine may lead to the dysregulation of metabolic, regulatory, and antioxidant pathways which result in cancer development (46). We also observed higher nitrotyrosine concentration in patients with tumour’s diameter >5 cm, in patients with tumours in T3 and T4 stage, in patients with lymph node and distant metastasis as well as in patients with diffuse type of gastric cancer which present with neural invasion. It can be concluded that higher nitrotyrosine levels are associated with development of penetrating deeper layers and more aggressive types of cancer, as well as metastasis and neural invasion.

### Protein glycation and glycoxidation

Overproduction of reactive oxygen and nitrogen species also result in oxidation and glycation of proteins, and combination of these processes is often called glycoxidation. In our work we observed decreased levels of tryptophan and increased levels of N-formylkynurenine, kynurenine, dityrosine, Amadori products and AGE in patients with gastric cancer in comparison with the healthy control. It is known that kynurenine and N-formylkynurenine formation depends on IDO (indoleamine 2,3-dioxygenase) activity – an enzyme responsible for tryptophan conversion to N-formylkynurenine. Increased activity of IDO results in the tryptophan depletion and consequently accumulation of its metabolites (N-formylkynurenine, kynurenine) which may lead to a strong inhibitory effect on the development of immune responses (47). It may be caused by enhanced activation of the enzyme and intensified tryptophan consumption by cancer cells. Recent studies performed on tumour models showed that IDO inhibition and thereby decreased formation of tryptophan metabolites could significantly enhance the antitumour activity of various chemotherapeutic and immunotherapeutic agents (48). Choi et al. (49) also observed significant differences in the level of tryptophan and its metabolites between patients with gastric cancer and control group, both in serum and gastric juice and they suggest that these biomarkers might be used to monitor gastric cancer. Tryptophan levels were lower in patients with tumours in G3 stage, T3 and T4, with lymph node and distant metastasis as well as in patients with neural and vascular invasion whereas kynurenine and N-formylkynurenine were increased in patients with lymph node metastasis. Dityrosine levels was higher in patients with distant metastasis. These observations may indicate the rapid catabolism of tryptophan in more advanced tumours, with a possible accumulation of subsequent products – N-formylkynurenine and kynurenine. Clinical studies have demonstrated that tryptophan metabolites promote tumour progression through modulation of the immunosuppressive microenvironment by multiple mechanisms (15). Studies performed on lung and esophageal cancers showed that aberrant tryptophan metabolism is associated with TNM stage and the degree of malignancy of tumours, which further affects prognosis (50).

We also observed increased concentration of N-formylkynurenine and kynurenine in GC patients with *Helicobacter pylori* infection. It seems that increased levels of N-formylkynurenine and kynurenine may be caused by enhancement of the IDO activity. In the immune response mechanism of infection, IDO activity is considered to be the first-line of defence against invading cells and may mediate an antimicrobial effect. Moreover, in the first phase of infection, IDO-mediated tryptophan depletion has mainly an antibacterial effect while in the later phase, it is an inhibitor of T-cell growth (51). Thus the presence of *Helicobacter pylori* may enhance the immune tolerance against cancer cells (47). Summarising, significantly higher kynurenine and reduced NO levels in patients with gastric cancer with *Helicobacter pylori* infection suggest that *Helicobacter pylori* may support immune tolerance leading to carcinogenesis.

Protein glycation products are Amadori products and advanced glycation end products (AGE). AGE have a strong affinity for the receptor for advanced glycation end-products (RAGE). Binding AGE to the RAGE may promote an initiation and activation of downstream signalling pathways including nuclear factor kappa B (NF-κB), p38 mitogen-activated protein kinesis (MAPK) or tumour necrosis factor–α (TNF-α). They take part in stimulating cell-proliferating growth factors and suppress endogenous autoregulatory functions leading to inflammation, localised tissue damage and cancer development (52). It has been discovered that upregulation of RAGE expression is associated with poor clinicopathological characteristics and poor overall survival as well as with invasion and metastasis indicating that RAGE may contribute to the malignant potential of GC (53, 54). Higher levels of AGE and Amadori products in patients with gastric cancer may be caused by anaerobic metabolism of glucose in cancer cells. Tumours present with an increased glucose uptake and a high rate of glycolysis (55). In our study, AGE and Amadori products were increased in III+IV types of gastric cancer according to Goseki classification. Amadori products were also higher in patients with tumour’s diameter >5 cm, with adenocarcinoma mucinosum and in T3 and T4 stage and present distant metastasis. This is not surprising because glycooxidative-modified proteins may form aggregates resistant to degradation by proteolytic enzymes. This favour the accumulation of modified proteins in cells and result in a gradual loss of their structure and biological function (27). Thus, it seems that the development of advanced cancer is associated with disturbances in the protein degradation process and their enhanced accumulation. Studies performed on renal cell carcinoma demonstrated that AGE *via* NF-κB activation may favour tumour cell proliferation, apoptosis inhibition, increases the tumour angiogenesis ability and the potential of tumour cell invasion and metastasis (56).

### Limitations

It has to be mentioned that there are limitations regarding our study. We had no information about the diet and physical activity of patients, therefore these factors were not included in the study. Although the sample size was quantified statistically, the study group was relatively small. Therefore, further studies on a larger group of gastric cancer patients are needed. The concentration of redox parameters was assessed only in blood samples (serum/plasma), giving our results an approximate value. Their level should also be assessed in postoperative tumour tissues and evaluation of blood-tissue correlations would then be advisable. Because we measured only the selected nitrosative stress/protein glycation biomarkers, we cannot fully explain the redox impairment in patients with gastric cancer. Additionally, we did not assess the concentration of kynurenine pathway metabolites, but only their fluorescence, which is also a strong limitation of our study. However, our work also has evident strengths. These include the careful selection of a study and control group without comorbidities. In addition, we were the first to assess the diagnostic utility of nitrosative stress/protein glycation biomarkers in respect to histopathological parameters, presence of *Helicobacter pylori* infection as well as presence of vascular or neural invasion and desmoplasia.

### Summary

To summarise, we have shown that the levels of circulating nitrosative stress markers (NO, S-nitrosothiols, nitrotyrosine) and glycoxidation products (kynurenine, N-formylkynurenine, dityrosine, AGE, Amadori products) were significantly increased in patients with gastric cancer. Although further research on a tissue model is needed, our results suggested that nitrosative stress and protein glycoxidation may play a significant role in gastric cancer development. We also showed that the levels of NO, kynurenine and N-formylkynurenine differ significantly between gastric cancer patients with and without *Helicobacter pylori* infection. Therefore, the next step is to determine the causal relationship between *Helicobacter pylori* and nitrosative stress/protein glycation in the development of gastric cancer. Most determined redox biomarkers differentiate with high sensitivity and specificity patients with gastric cancer from a healthy population.

This study may be the first step to a further, more advanced analysis using molecular methods concentrating on the assessment of the diagnostic utility of redox markers in a larger group of gastric cancer patients. It also seems advisable to evaluate the association between the intensity of nitrosative stress and the survival of patients with gastric cancer.

## Data availability statement

The original contributions presented in the study are included in the article/**Supplementary Material**. Further inquiries can be directed to the corresponding author.

## Ethics statement

The studies involving human participants were reviewed and approved by Bioethics Committee of the Medical University of Bialystok, Poland (permission number APK-002/238/2022). The patients/participants provided their written informed consent to participate in this study.

## Author contributions

Conceptualization, JD; Methodology, JD and MM; Software, JD, MM, and PŻ; Validation, JD, MM, and PŻ; Formal Analysis, JD, MM, and AP; Investigation, JD, MM, and AZ; Resources, KZ and AP; Data Curation, JD and MM; Writing – Original Draft Preparation, JD; Writing – Review and Editing, JD, MM, AZ, and JM-K; Visualization, JD and AP; Supervision, JD and MM; Project Administration, JD and MM; Funding Acquisition, JD, MM, and JM-K. All authors contributed to the article and approved the submitted version.
