# Supplementary material for: Could circulating biomarkers of nitrosative stress and protein glycoxidation be useful in patients with gastric cancer?
Source: Front Oncol. 2023 Jul 12;13:1213802. doi: 10.3389/fonc.2023.1213802 (PMC10369187; doi:10.3389/fonc.2023.1213802)
Supplement: Supplementary file 1 [file DataSheet_1.docx]

Supplementary File 1. Comparison of nitrosative stress parameters and glycoxidation products between men and women with gastric cancer. The data are presented as median (minimum - maximum). Abbreviations: NO – nitric oxide, AGE – advanced glycation end products.
