# Supplementary material for: Could circulating biomarkers of nitrosative stress and protein glycoxidation be useful in patients with gastric cancer?
Source: Front Oncol. 2023 Jul 12;13:1213802. doi: 10.3389/fonc.2023.1213802 (PMC10369187; doi:10.3389/fonc.2023.1213802)
Supplement: Supplementary file 2 [file DataSheet_2.docx]

|  | **Control group** | **Gastric cancer** | **p-value** |
| --- | --- | --- | --- |
| NO | 0.3382 (0.2230-1.195) | 0.7932 (0.4686-2.836) | <0.0001 |
| S-nitrosothiols | 0.3468 (0.2138-0.4516) | 0.7526 (0.2122-1.017) | <0.0001 |
| Nitrotyrosine | 165.4 (95.86-259.5) | 1215 (568.2-2599) | <0.0001 |
| Tryptophan | 1499 (1120-2121) | 2081 (816-2463) | <0.0001 |
| Kynurenine | 869.7 (513.7-1305) | 1523 (297.7-2329) | <0.0001 |
| N-formylkynurenine | 684 (426.8-911.3) | 957 (438.6-1677) | <0.0001 |
| Dityrosine | 557.1 (396.1-783.7) | 1308 (776.7-1621) | <0.0001 |
| Amadori products | 8.679 (6.080-11.01) | 34.26 (21.36-38.31) | <0.0001 |
| AGE | 130.6 (79.01-198.1) | 879.6 (469.6-1295) | <0.0001 |
|  | **Women** | **Men** |  |
| NO | 0.8132 (0.4686-1.997) | 0.7533 (0.4786-2.836) | 0.6265 |
| S-nitrosothiols | 0.7248 (0.3470-0.8670) | 0.7530 (0.2122-1.017) | 0.8360 |
| Nitrotyrosine | 880 (568.2-1661) | 1246 (608.3-2387) | 0.0686 |
| Tryptophan | 21.94 (15.67-24.58) | 20.68 (14.24-24.63) | 0.7468 |
| Kynurenine | 1754 (311.5-3329) | 1422 (244-2643) | 0.0555 |
| N-formylkynurenine | 1038 (124.8-2262) | 864.5 (100.7-1677) | 0.0759 |
| Dityrosine | 1389 (1047-1639) | 1292 (789.3-1499) | 0.0640 |
| Amadori products | 34.14 (23.42-79.67) | 34.26 (21.36-67.88) | 0.6044 |
| AGE | 133.4 (73.04-238.3) | 117.7 (56.44-184.1) | 0.0759 |
| **Tumour size** | **≤ 5cm** | **> 5cm** |  |
| NO | 0.6035 (0.4686-1.836) | 0.7458 (0.5136-2.391) | 0.0180 |
| Nitrotyrosine | 779.9 (568.2-1867) | 1358 (722.7-2599) | 0.0216 |
| Amadori products | 29.75 (23.81-53.72) | 36.79 (27.85-79.67) | 0.0078 |
| **Histological type** | **Adenocarcinoma** | **Adenocarcinoma mucinosum** |  |
| NO | 0.6234 (0.4686-1.902) | 1.652 (0.6684-2.836) | <0.0001 |
| Amadori products | 31.49 (21.36-36.95) | 35.40 (23.42-79.67) | 0.0405 |
| **Histological differentiation grade** | **G2** | **G3** |  |
| S-nitrosothiols | 68.39 (47.83-78.43) | 76.35 (21.22-101.7) | 0.0214 |
| Tryptophan | 2236 (1424-2463) | 1988 (653.3-2264) | 0.0271 |
| **Helicobacter pylori infection** | **Absent** | **Present** |  |
| NO | 1.997 (0.4786-2.836) | 0.7633 (0.5136-1.752) | 0.0265 |
| Kynurenine | 1528 (1028-2249) | 1834 (1350-3329) | 0.0442 |
| N-formylkynurenine | 959.1 (571.1-1160) | 1280 (807.9-2262) | 0.0345 |
| **Depth of tumour invasion** | **T1+T2** | **T3+T4** |  |
| Nitrotyrosine | 791.3 (608.3-1867) | 1295 (568.2-2599) | 0.0198 |
| Tryptophan | 2236 (1428-2463) | 1939 (653.3-2339) | 0.0201 |
| Amadori products | 30.06 (21.36-43.12) | 34.34 (27.85-79.67) | 0.0246 |
| **Lymph node metastasis** | **N0** | **N1+N2** |  |
| Nitrotyrosine | 751.3 (608.3-1358) | 1206 (568.2-2599) | 0.0074 |
| S-nitrosothiols | 70.09 (34.70-84.61) | 75.30 (52.70-101.70) | 0.0424 |
| Tryptophan | 2236 (1798-2463) | 1864 (653.3-2264) | 0.0325 |
| N-formylkynurenine | 804.9 (100.7-1280) | 958.6 (571.1-2262) | 0.0410 |
| **Distant metastasis** | **M0** | **M1+M2** |  |
| Nitrotyrosine | 882.8 (568.2-1924) | 1300 (694.1-2599) | 0.0318 |
| S-nitrosothiols | 70.52 (34.70-84.61) | 78.26 (21.22-101.70) | 0.0065 |
| Tryptophan | 2275 (1937-2463) | 2035 (816-2397) | 0.0178 |
| Dityrosine | 1246 (807-1476) | 1384 (954.7-1888) | 0.0218 |
| Amadori products | 26.58 (21.36-36.95) | 34.26 (24.05-79.67) | 0.0144 |
| **Lauren’s classification** | **Intestinal** | **Diffuse** |  |
| NO | 0.6184 (0.4686-1.902) | 1.178 (0.5335-2.601) | 0.0058 |
| Nitrotyrosine | 922.9 (568.2-2107) | 1409 (694.1-2599) | 0.0606 |
| **Goseki classification** | **I+II** | **III+IV** |  |
| NO | 0.7083 (0.4786-1.692) | 1.093 (0.4686-2.836) | 0.0404 |
| S-nitrosothiols | 67.57 (34.70-84.61) | 75.22 (63.48-101.70) | 0.0201 |
| Tryptophan | 2194 (1590-2397) | 1905 (653.3-2463) | 0.0194 |
| Kynurenine | 1420 (244-1834) | 1596 (1028-3329) | 0.0479 |
| N-formylkynurenine | 804.9 (100.7-1280) | 999.4 (582.2-2262) | 0.0110 |
| Amadori products | 29.43 (21.36-41.77) | 34.26 (23.81-44.46) | 0.0307 |
| AGE | 114.7 (59.96-157.1) | 134.2 (101.9-238.3) | 0.0083 |
| **Vascular invasion** | **Absent** | **Present** |  |
| Tryptophan | 2169 (1428-2463) | 1864 (653.3-2189) | 0.0328 |
| **Neural invasion** | **Absent** | **Present** |  |
| Nitrotyrosine | 757.0 (608.3-1197) | 1203 (568.2-2599) | 0.0259 |
| Tryptophan | 2139 (816-2463) | 1864 (653.3-2264) | 0.0489 |

Supplementary File 2. Comparison of chosen parameters of nitrosative stress and glycoxidation products between patients with gastric cancer and the control group, between women and men with gastric cancer and in groups of patients with gastric cancer categorized by histopathological parameters.
